# Genome-Wide Differences in DNA Methylation Changes in Two Contrasting Rice Genotypes in Response to Drought Conditions

**DOI:** 10.3389/fpls.2016.01675

**Published:** 2016-11-08

**Authors:** Wensheng Wang, Qiao Qin, Fan Sun, Yinxiao Wang, Dandan Xu, Zhikang Li, Binying Fu

**Affiliations:** ^1^Institute of Crop Sciences/National Key Facility for Crop Gene Resources and Genetic Improvement, Chinese Academy of Agricultural SciencesBeijing, China; ^2^College of Agronomy, Anhui Agricultural UniversityHefei, China; ^3^Shenzhen Institute for Innovative Breeding, Chinese Academy of Agricultural SciencesShenzhen, China

**Keywords:** DNA methylation, rice, drought stress, epigenetics, MeDIP-seq

## Abstract

Differences in drought stress tolerance within diverse rice genotypes have been attributed to genetic diversity and epigenetic alterations. DNA methylation is an important epigenetic modification that influences diverse biological processes, but its effects on rice drought stress tolerance are poorly understood. In this study, methylated DNA immunoprecipitation sequencing and an Affymetrix GeneChip rice genome array were used to profile the DNA methylation patterns and transcriptomes of the drought-tolerant introgression line DK151 and its drought-sensitive recurrent parent IR64 under drought and control conditions. The introgression of donor genomic DNA induced genome-wide DNA methylation changes in DK151 plants. A total of 1190 differentially methylated regions (DMRs) were detected between the two genotypes under normal growth conditions, and the DMR-associated genes in DK151 plants were mainly related to stress response, programmed cell death, and nutrient reservoir activity, which are implicated to constitutive drought stress tolerance. A comparison of the DNA methylation changes in the two genotypes under drought conditions indicated that DK151 plants have a more stable methylome, with only 92 drought-induced DMRs, than IR64 plants with 506 DMRs. Gene ontology analyses of the DMR-associated genes in drought-stressed plants revealed that changes to the DNA methylation status of genotype-specific genes are associated with the epigenetic regulation of drought stress responses. Transcriptome analysis further helped to identify a set of 12 and 23 DMR-associated genes that were differentially expressed in DK151 and IR64, respectively, under drought stress compared with respective controls. Correlation analysis indicated that DNA methylation has various effects on gene expression, implying that it affects gene expression directly or indirectly through diverse regulatory pathways. Our results indicate that drought-induced alterations to DNA methylation may influence an epigenetic mechanism that regulates the expression of unique genes responsible for drought stress tolerance.

## Introduction

Drought is the most serious environmental stress limiting crop growth, development, and yield (Farooq et al., 2009). Rice is sensitive to drought stress, and the effects of drought on rice plants vary among genotypes and developmental stages (Datta et al., 1988; Degenkolbe et al., 2009). The genes responsible for the mechanisms underlying rice drought tolerance have been studied for decades. Drought tolerance is a complex trait involving several genetic pathways (Todaka et al., 2015). However, little is known about the role of epigenetic processes during the molecular regulation of rice drought tolerance.

DNA methylation is an epigenetic modification that is important for plant growth and development as well as responses to environmental stresses (Bender, 2004; Meyer, 2015). As one of the earliest detected epigenetic modifications, DNA methylation has been observed in many organisms. The methylation of eukaryotic DNA frequently occurs at the 5 position of cytosine, yielding 5-methylcytosine. Under normal growth conditions, the proportion of cytosines that are methylated in plants is 20–30% (Finnegan et al., 1998). There is much evidence indicating that changes to DNA methylation considerably affect the ability of plants to respond to environmental stresses (Boyko and Kovalchuk, 2008). Salt-responsive genes are differentially methylated under salt stress conditions, indicating that DNA methylation influences plant responses to environmental stress (Wang M. et al., 2014). Furthermore, altered DNA methylation in response to salt stress is organ- and genotype-specific, and is not directly associated with salt tolerance (Wang et al., 2011b; Karan et al., 2012). Drought stress also induces genome-wide changes to DNA methylation, leading to altered gene expression levels (Wang et al., 2011a; Tang et al., 2014). These observations suggest that DNA methylation influences abiotic stress responses and adaptations.

DNA methylation was first analyzed by high-performance liquid chromatography to assess global DNA methylation changes (Cai and Chinnappa, 1999). However, this technique did not allow the determination of the methylation status at individual gene loci. Methylation-sensitive amplified polymorphism (MSAP) is a modified version of the amplified fragment length polymorphism technique, and was developed to assess the status of cytosine methylation in GGCC genomic fragments (Xiong et al., 1999). However, this method can detect only a few methylated sequences because of the limitations of the isoschizomers used. New sequencing-based technologies to analyze genome-wide DNA methylation, including bisulfite sequencing and methylated DNA immunoprecipitation sequencing (MeDIP-seq), are now commonly used (Li et al., 2010; Taiwo et al., 2012). Bisulfite sequencing was recently used to profile diverse methylomes among cultivated and wild rice lines (Li et al., 2012), analyze DNA methylation patterns in different rice genotypes (Garg et al., 2015) and investigate the DNA methylation changes in pesticide-treated rice plants (Lu et al., 2016). The MeDIP-seq platform has been applied to characterize DNA methylation status during rice seed development (Xing et al., 2015) and fertility transitions of the photoperiod- and thermo-sensitive male sterile line (Hu et al., 2015). However, little is known about the effects of genome-wide cytosine methylation on drought responses in rice plants.

In a previous study, we assessed DNA methylation changes in a drought-tolerant rice line (DK151) and its drought-sensitive recurrent parent (IR64) using the MSAP technique. Many genome site-specific methylation differences were detected in both genotypes under drought stress conditions. Drought-induced DNA demethylation/methylation patterns in rice may be important for adaptation to drought conditions (Wang et al., 2011a). However, most of the DNA methylation of non-CCGG genomic sites was not detected because of the limitations of the MSAP technique. In the current study, to explore genome-wide changes to cytosine methylation in rice lines DK151 and IR64 under drought stress conditions, we used MeDIP-seq to characterize differentially methylated regions (DMRs). We also investigated the effects of DMRs on gene expression using microarrays. The results of this study may help to clarify the epigenetic mechanisms that regulate drought stress tolerance in rice plants.

## Materials and Methods

### Rice Materials and Growth Conditions

According to our previous study, DK151 showed significantly improved tolerance to drought over IR64 (Wang et al., 2011a). Further, DK151 showed significantly better performance than IR64 under drought stress conditions (Supplementary Figure S1). Seeds of the drought-tolerant DK151 rice line and its drought sensitive-recurrent parent IR64 were surface-sterilized and germinated at 37°C in distilled water for 2 days. The germinated seeds were then transferred to a seedling nursery. Rice plants at the four-leaf stage were transplanted into plastic pots (height: 30 cm; diameter: 35 cm) filled with sterilized field soil. Seedlings were grown in a greenhouse at 29/22°C day/night temperatures at the Institute of Crop Sciences of the Chinese Academy of Agricultural Sciences (Beijing, China). Two healthy seedlings from each genotype were planted in one pot (equidistant from each other). Six replicates (three for MeDIP-seq and three for microarray analysis) were prepared for the drought stress and control conditions.

Drought stress was simulated by withholding water at the tillering stage as previously described by Huang et al. (2014). Well-watered plants were used as controls. The three uppermost leaves were harvested from each sample 3 days after initiating the drought treatment. All samples were immediately frozen in liquid nitrogen and stored at -80°C.

### Genomic DNA Extraction and Methylated DNA Immunoprecipitation Sequencing

Genomic DNA was extracted from drought-stressed and control DK151 and IR64 plants using the AxyPrep Multisource Genomic DNA Miniprep Kit (Axygen Biosciences). Each sample consisted of three biological replicates. Libraries prepared using non-immunoprecipitated “input” DNA from three biological replicates were sequenced as controls. The MeDIP-seq DNA libraries were prepared according to the protocol described by Li et al. (2010). Briefly, 5 μg DNA from each sample was sonicated to produce DNA fragments (100–500 bp). Libraries were then constructed using the Paired-End DNA Sample Prep Kit (Illumina, San Diego, CA, USA). Adapter-ligated DNA was immunoprecipitated using a monoclonal anti-methylcytidine antibody. The specificity of the enrichment was confirmed by quantitative reverse transcription polymerase chain reaction (PCR) using the SYBR Green Master Mix (Applied Biosystems). The enriched methylated fragments and input DNA were purified with DNA Clean & Concentrator-5 columns (Zymo). The purified DNA was then used as the template for adapter-mediated PCR. Amplicon quality and quantity were evaluated using the 2100 Analyzer DNA 1000 chips (Agilent). Ultra-high-throughput 50PE sequencing was conducted using the Illumina HiSeq 2000 system. Raw sequencing data were processed by the Illumina base-calling pipeline.

### Methylated DNA Immunoprecipitation Sequencing Data Analysis

Sequencing adapters were removed and low-quality bases (quality < 20) were trimmed from the 5′ and 3′ ends of reads using an in-house Perl script. The obtained clean reads were then mapped to the rice reference genome (IRGSP-1.0/MSU7) using the default parameters of the BWA program (version 0.7.12). The mapped high-quality reads were used for genome-wide distribution analyses (e.g., distribution among chromosomes and in diverse components of the rice genome).

Model-based analysis of MeDIP-seq data (Zhang et al., 2008) was used to detect highly methylated regions (peaks), and the peak locations were summarized with a Perl script. The Picard program^1^ converted mapping results to the BAM format. We used the Bioconductor package MEDIPS (Lienhard et al., 2014) to identify DMRs among the different samples (bin size: 200 bp; adjusted *p*-value < 0.1 for edgeR). The DMRs with a more than twofold change in read counts were classified as hyper- or hypo-methylated regions.

### Bisulfite Sequencing of Differentially Methylated Genes

The differentially methylated gene regions were confirmed by Sanger sequencing. For each target region, primers were designed (Supplementary Table S1). Bisulfite conversions were completed using the EZ DNA Methylation-Gold Kit (Zymo). Briefly, 1.2 μg DNA was mixed with the C–T conversion reagent, and then incubated at 98°C for 10 min, 64°C for 2.5 h, and 4°C for 30 min. Modified DNA was purified and stored at -20°C. We then amplified fragments (approximately 400 bp). For each PCR, 2.0 μl bisulfite-treated DNA was used in a 20-μl reaction volume. Purified amplicons were cloned into the pEASY-T5 vector (TransGen, Beijing, China) and sequenced. At least 18 clones were sequenced and analyzed for each sample. Methylation rates were expressed as the proportion (%) per site for each of the three types of cytosines (CG, CHG, and CHH). Methylation rates were calculated by dividing the number of non-converted (methylated) cytosines by the total number of cytosines within the assay. The bisulfite sequencing data were analyzed using the Kismeth online tool^2^.

### RNA Isolation and Microarray Analysis

Total RNA was extracted from the leaves of drought-stressed and control DK151 and IR64 plants according to the GeneChip Expression Analysis Technical Manual (Affymetrix). Briefly, total RNA was extracted from frozen leaf tissue using TRIzol reagent and then purified and concentrated using the RNeasy MinElute Cleanup Kit (Qiagen, Germany). CapitalBio Corporation (Beijing) then completed the following steps. Double-stranded cDNA was synthesized from 2 μg total RNA. Additionally, biotin-tagged cRNA was generated in an *in vitro* transcription reaction, which was carried out using the MessageAmp II aRNA Amplification Kit. The resulting cRNA was fragmented (35–200 bases) according to the GeneChip Expression Analysis Technical Manual, and then hybridized to a rice genome array (Affymetrix) containing 48,564 *japonica* and 1,260 *indica* sequences. Samples were rotated at 45°C for 16 h in the GeneChip Hybridization Oven 640 (Affymetrix). The chips were then washed and stained in the GeneChip Fluidics Station 450 (Affymetrix), and then scanned using the GeneChip Scanner 3000 (Affymetrix).

Differentially expressed genes (DEGs) (i.e., drought-stressed vs. control samples; genotype vs. genotype) were identified using the two-class unpaired comparison method in the Significant Analysis of Microarray program (criteria: >2-fold change and significant *q*-value [false discovery rate-adjusted *p*-value] < 0.05 based on three independent biological replicates).

To validate the results of transcriptome analysis, a subset of DEGs were verified using qRT-PCR. PCR was conducted according to the methods described by Swarbrick et al. (2008). The sequences of the selected genes were downloaded from the MSU Rice Genome Annotation Project (Kawahara et al., 2013) and used to design primers with Primer 5^3^ (Supplementary Table S2). We performed three biological repetitions for each experiment. 49 genes were tested in 50-μl reactions using the SYBR^®^ Green PCR Master Mix kit (Applied Biosystems, Carlsbad, CA, USA) following the manufacturer’s protocol.

## Results

### Methylated DNA Immunoprecipitation Sequencing Analysis

The MeDIP-seq libraries were constructed using DNA extracted from drought-stressed DK151 and IR64 samples (i.e., DK151s and IR64s) and control samples (i.e., DK151c and IR64c), and then subjected to high-throughput Illumina sequencing. A total of 21,311,666–21,925,622 raw reads were generated for the four samples. After low-quality data were removed, approximately 85% of the reads were assessed as clean data and were further analyzed and mapped. An average of approximately 11 million unique mapped reads were obtained for the four samples following a high-quality read alignment against the rice genome (**Table 1**).

**Table 1:** Summary of MeDIP-seq results for IR64 and DK151 under drought (s) and control (c) conditions.

Sample	Total reads	Mapped reads	Mapped reads in total reads (%)	Unique mapped reads	Unique mapped reads (%)
IR64c	21,925,622	18,612,227	84.89	11,138,020	50.80
IR64s	21,311,666	18,255,523	85.58	10,960,305	50.20
DK151c	21,879,742	18,540,441	84.92	11,297,601	51.55
DK151s	21,662,524	18,502,367	85.42	11,149,720	51.47

**FIGURE 1:**
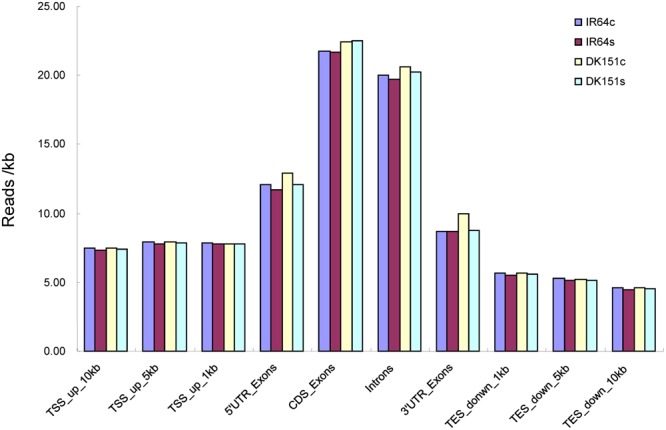
**MeDIP-seq read distribution in the genomic regions of DK151 and IR64 under drought stress (s) and control (c) conditions.** All uniquely mapped reads upstream of the transcription start site (TSS), 5’UTR, exons, and introns and downstream of the transcription termination site (TES) were calculated and normalized as reads per kilobase (reads/kb).

The normalized data (reads/kb) for the unique mapped reads were further analyzed to characterize the genomic distribution of reads (i.e., upstream of the transcription start site, gene body region, and downstream of the transcription termination site). The MeDIP-seq reads were detected more often in the gene body regions than in the 5′ and 3′ flanking regions (**Figure 1**), indicating the methylation rate was highest in the gene body regions.

### Differential DNA Methylation in DK151 and IR64 under Drought Stress and Control Conditions

The model-based analysis of MeDIP-seq data detected 27,888 (DK151s), 29,764 (IR64s), 26,189 (DK151c), and 28,515 (IR64c) methylation peaks, indicating there was a greater abundance of DNA methylation peaks in the IR64 line than in the DK151 line (**Table 2**). The length of the methylation peaks ranged from 200 to 3,500 bp, with an average of 2,032 to 2,202 bp in the four samples. Additionally, the total length of the methylation peaks covered approximately 16% of the whole rice genome (Supplementary Figure S2; Supplementary Table S3). Overall, the peaks were differentially distributed among genomic components, with the distal intergenic regions containing the most methylation peaks (about 35%), followed by the promoter regions. Within the gene body regions, the methylation peaks were most often detected in the first exon (**Table 2**).

**Table 2:** Distribution of methylation peaks in different genomic components.

Genomic component	IR64c	IR64s	DK151c	DK151s
Promoter (2–3 kb)	4437 (15.56%)	4667 (15.68%)	3923 (14.98%)	4289 (15.38%)
Promoter (1–2 kb)	3949 (13.85%)	4259 (14.31%)	3237 (12.36%)	3684 (13.21%)
Promoter (< = 1 kb)	1748 (6.13%)	1938 (6.51%)	1587 (6.06%)	1760 (6.31%)
5′ UTR	2427 (8.51%)	2452 (8.24%)	2514 (9.6%)	2432 (8.72%)
First exon	2575 (9.03%)	2536 (8.52%)	2642 (10.09%)	2605 (9.34%)
Other exon	151 (0.53%)	173 (0.58%)	134 (0.51%)	142 (0.51%)
First intron	63 (0.22%)	60 (0.2%)	39 (0.15%)	53 (0.19%)
Other intron	140 (0.49%)	149 (0.5%)	110 (0.42%)	128 (0.46%)
3′ UTR	510 (1.79%)	533 (1.79%)	490 (1.87%)	499 (1.79%)
Downstream (< = 3 kb)	2404 (8.43%)	2464 (8.28%)	2153 (8.22%)	2342 (8.4%)
Distal intergenic	10111 (35.46%)	10533 (35.39%)	9360 (35.74%)	9954 (35.69%)
Total	28515 (100%)	29764 (100%)	26189 (100%)	27888 (100%)

**FIGURE 2:**
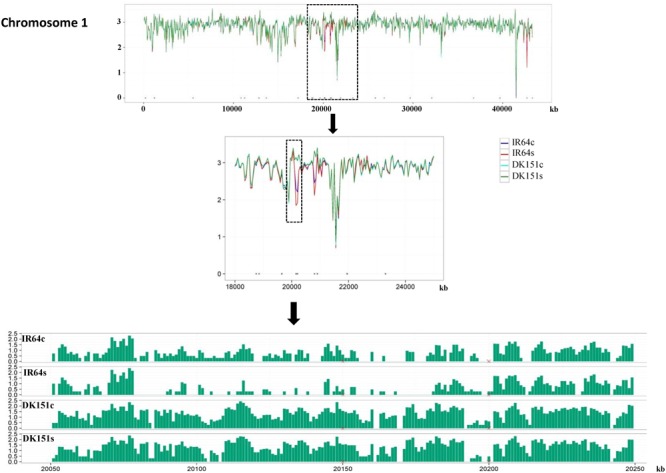
**Differential DNA methylation patterns of a region on Chromosome 1 in IR64 and DK151 under drought stress (s) and control (c) conditions**.

**FIGURE 3:**
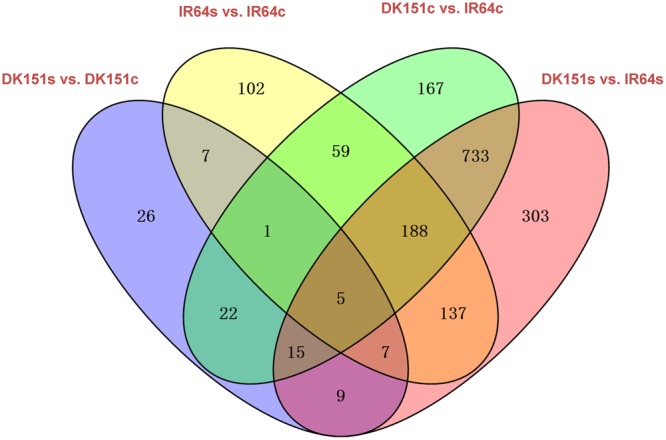
**Comparison of differentially methylated regions in four pairs of samples, DK151s vs. DK151c, IR64s vs. IR64c, DK151c vs. IR64c, and DK151s vs. IR64s**. DK151s, DK151c, IR64s, and IR64c indicate DK151 and IR64 under drought stress (s) and control (c) conditions, respectively.

The methylation peaks in the four samples were compared and summarized in a Venn diagram. More than 70% of the peaks were common to all four samples, indicating that most of the DNA methylation peaks are relatively stable between the two genotypes under control and drought stress conditions. In contrast, approximately 2–5% of the peaks were unique to specific samples (Supplementary Figure S2). Examples of the DNA methylation status of the four samples as determined by MeDIP-seq are presented in **Figure 2**. The DMRs were further analyzed (criteria: >2-fold changes and *q*-value < 0.05), and we detected 506 (IR64s vs. IR64c), 92 (DK151s vs. DK151c), 1,190 (DK151c vs. IR64c), and 1,397 (DK151s vs. IR64s) DMRs (**Table 3**; **Figure 3**).

**Table 3:** Differentially methylated regions (DMRs) detected between samples.

Comparison	Hyper-methylated DMRs	Hypo-methylated DMRs	Total
IR64s vs. IR64c	336	170	506
DK151s vs. DK151c	48	44	92
DK151c vs. IR64c	500	690	1190
DK151s vs. IR64s	578	819	1397

### Differentially Methylated Regions between DK151 and IR64 under Control and Drought Stress Conditions

Under normal growth conditions, 500 and 690 DMRs were hyper- and hypo-methylated, respectively, in DK151c plants compared with IR64c plants (**Table 3**). These DMRs were randomly distributed among the 12 rice chromosomes, and only 125 were located in introgressed regions (Supplementary Table S4), indicating that backcross introgressions resulted in genome-wide DNA methylation changes in the DK151 line. We identified 397 and 556 genes associated with these hyper- and hypo-methylated DMRs, respectively (Supplementary Table S4). Gene ontology (GO) enrichment analysis using agriGO (Du et al., 2010) revealed that the hypo-methylated DMR-associated genes were mainly related to stress response, programmed cell death, and nutrient reservoir activity, while the hyper-methylated DMR-associated genes were mostly related to lyase activity and magnesium ion binding.

We detected 578 and 819 DMRs that were hyper- and hypo-methylated, respectively, in DK151s plants compared with IR64s plants, under drought stress conditions. Most of these DMRs were also observed in the comparison between DK151c and IR64c samples (**Figure 3**), suggesting that the majority of DNA methylation sites were unaffected by drought stress. These observations are consistent with the analysis of DNA methylation peaks.

### Differentially Methylated Regions in DK151 and IR64 Induced by Drought Stress

**FIGURE 4:**
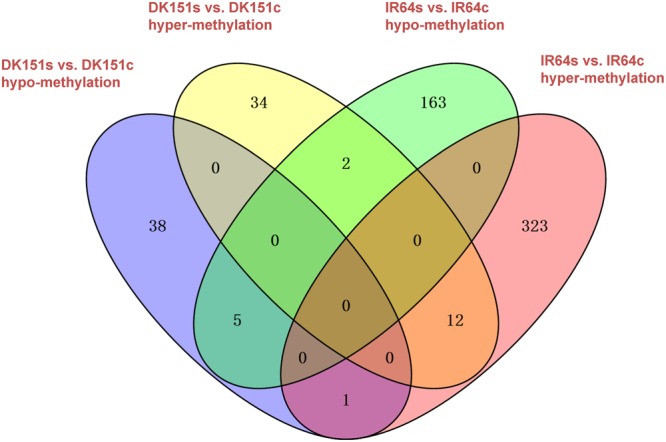
**Venn diagram comparing hyper- or hypo-methylated regions in two pairs of samples, DK151s vs. DK151c, IR64s vs. IR64c.** DK151s, DK151c, IR64s, and IR64c indicate DK151 and IR64 under drought stress (s) and control (c) conditions, respectively.

We identified 48/44 and 336/170 DMRs that were hyper-/hypo-methylated in DK151 and IR64, respectively, under drought stress conditions (relative to the levels in control samples) (**Table 3**). Overall, there were more hyper-methylated DMRs than hypo-methylated DMRs in both genotypes, implying that hyper-methylation is prevalent in responses to drought stress. Additionally, more DMRs were detected in the drought-sensitive IR64 genotype than in the drought-tolerant DK151 line, with most of these DMRs being genotype-specific (**Figure 4**). This suggests that DNA methylation changes are highly associated with drought stress responses. Furthermore, the fact that fewer DMRs were observed in the drought-tolerant DK151 than in the drought-sensitive IR64 implies that DNA methylation is more stable in the drought-tolerant genotype.

We identified 48/44 genes associated with the hyper-/hypo-methylated DMRs in the DK151s vs. DK151c comparison (Supplementary Table S5). GO analysis revealed that the 44 hypo-methylated genes were mostly related to transport, establishment of localization, and nucleotide binding. The genes included those encoding the transcription factors (TFs) TCP, NAC, and WRKY, as well as two cell wall-associated kinase genes (*OsWAK2* and *OsWAK3*), two *Osmotin-34* genes, and two *NB-ARC* genes. The 48 hyper-methylated genes were primarily related to stress responses, transport, and regulation of transcription. The proteins encoded by the hyper-methylated genes included two auxin-responsive factors, the AP2 TF, nuclear transport factor 2 (NTF2), two UDP-glucosyl transferases, and two ARM repeat superfamily proteins. Of the drought-induced DMRs in DK151, only eight (i.e., four hypo- and four hyper-methylated) were localized in introgressed intervals (Supplementary Table S5).

We detected 289/144 genes in the hyper-/hypo-methylated DMRs during the comparison between IR64s and IR64c samples (Supplementary Table S6). GO enrichment analysis results revealed that the 289 hyper-methylated genes were mostly related to programmed cell death, responses to stimuli, transferase activity, and electron carrier activity. In contrast, the 144 hypo-methylated genes were mainly associated with responses to stimuli and nutrient reservoir activity. These findings indicate that drought stress clearly affects the DNA methylation status of these genes in IR64 plants. The GO annotation results suggest that DNA methylation changes affect a diverse range of genes during drought stress responses in the two contrasting genotypes.

Twenty DMR-associated genes were common between the DK151s vs. DK151c and IR64s vs. IR64c comparisons. Most of these genes underwent the same DNA methylation changes in the two genotypes. However, LOC_Os02g29480 (NTF2), LOC_Os06g43590 (vacuolar protein sorting 46.1), and LOC_Os07g10580 (hydrophobic protein LTI6A) exhibited the opposite DNA methylation changes in drought-stressed DK151 and IR64 plants (Supplementary Tables S5 and S6). LOC_Os02g29480 and LOC_Os07g10580 were hypo-methylated (at the 5′ untranslated region) in DK151, but hyper-methylated in IR64. LOC_Os06g43590 was hyper-methylated (at the downstream region) in DK151, but hypo-methylated in IR64. These results indicate these genes are involved in genotype-specific responses to drought conditions.

**FIGURE 5:**
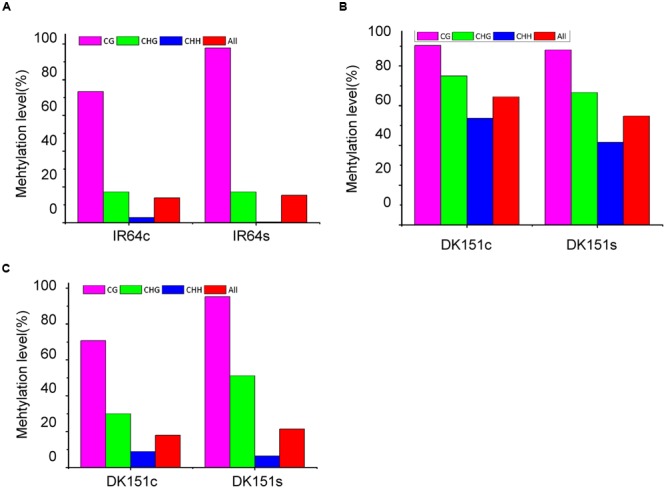
**Bisulfite sequencing of three DMR-associated genes. (A)** LOC_Os12g23260 (ureide permease); **(B)** LOC_Os02g29464 (DNA repair protein RAD50); **(C)** LOC_Os12g24020 (glycosyl hydrolase family 9 protein). The red, green and blue columns in the histograms refer to the collective methylation levels (percentages) of CG, CHG, and CHH, respectively.

To confirm the accuracy of the DMR results obtained by MeDIP-seq, several DMR-associated genes underwent bisulfite sequencing, including LOC_Os02g29464 (DNA repair protein RAD50), LOC_Os12g23260 (ureide permease), and LOC_Os12g24020 (glycosyl hydrolase family 9 protein). The bisulfite sequencing data were consistent with the MeDIP-seq profiles. For example, LOC_Os02g23260 was hyper-methylated in IR64 under drought stress conditions, but no DNA methylation changes were detected in this gene in drought-stressed DK151 plants. In contrast, LOC_Os12g23260 and LOC_Os12g24020 were differentially methylated only in DK151 plants exposed to drought stress (**Figure 5**).

### Identification of Differentially Expressed Genes in DK151 and IR64 under Control and Drought Stress Conditions

To clarify the relationship between DNA methylation and gene expression, we generated transcriptome profiles for DK151 and IR64 using the Affymetrix rice microarray. Under normal growth conditions, we detected 3,295 and 2,346 up- and down-regulated genes, respectively, in DK151 plants compared with IR64 plants (**Table 4**; Supplementary Table S7). This result suggests there are gene expression differences between the two rice genotypes even in the absence of stress. A GO enrichment analysis revealed the DEGs were associated with a diverse range of functional categories, including regulation of transcription, regulation of cellular metabolic process, protein modification, transport, and protein binding (up-regulated genes), as well as translation, small molecular metabolic processes, cellular component biogenesis, and RNA metabolic processes (down-regulated genes).

**Table 4:** Differentially expressed genes detected between samples.

Comparison	Up-regulated	Down-regulated	Total
DK151c vs. IR64c	3295	2346	5641
IR64s vs. IR64c	1840	1236	3076
DK151s vs. DK151c	1721	1916	3637

**FIGURE 6:**
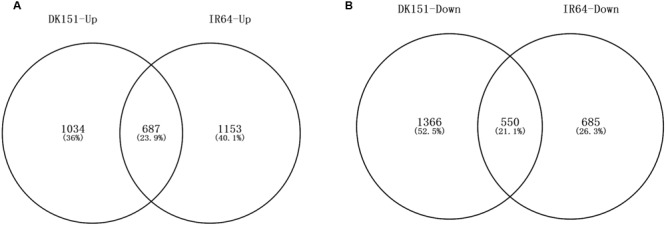
**Venn diagram of up-regulated **(A)** and down-regulated **(B)** genes in DK151 and IR64 under drought stress compared with respective controls.** Differentially expressed genes were identified by twofold changes and significant *q*-values (FDR adjusted *p*-values) less than 0.05 based on three independent biological replicates.

A comparison between the drought-stressed DK151 and IR64 plants and their respective controls revealed 3,636 (1,721 up-regulated and 1,915 down-regulated) and 3,075 (1,840 up-regulated and 1,236 down-regulated) DEGs in DK151 and IR64, respectively (**Table 4**; Supplementary Tables S8 and S9). Among these, 687 and 550 genes were commonly up- or down-regulated, respectively, in both genotypes (**Figure 6**). The DK151 up-regulated genes were mainly related to carbohydrate metabolic processes, catabolic processes, responses to abiotic stimuli, and transport, while the down-regulated genes were mostly associated with protein modification processes, transcription, regulation of metabolic processes, photosynthesis, and cellular protein metabolic processes. In contrast, the up-regulated IR64 genes were involved in carbohydrate metabolic processes, responses to abiotic stimuli, regulation of gene expression, and localization, while the down-regulated genes were associated with photosynthesis, carbohydrate metabolic processes, oxidation–reduction, transport, and membranes. These results indicate that many genes with diverse functions were involved in drought stress responses in DK151 and IR64 plants. Additionally, the genotype-specific GO categories for these DEGs revealed differences in the molecular mechanisms underlying drought stress responses, which were consistent with the contrasting drought tolerance phenotypes.

To validate the transcriptome results, qRT-PCR was used to independently assess expression levels for 49 genes of two genotypes (genes and primer sets are shown in Supplementary Table S2). The strong correlation (*R*^2^ = 0.8503) between transcript levels and qRT-RCR expression values indicate that there was good agreement between both approaches (Supplementary Figure S3).

### Correlation between Differentially Methylated Regions and Differentially Expressed Genes

To determine the influence of DNA methylation on gene expression, we first compared the DMRs and DEGs in DK151 with those in IR64 under normal growth conditions. Among the 5,641 DEGs (3,295 up-regulated and 2,346 down-regulated), 103 were located in DMRs, including 41 hyper- and 62 hypo-methylated regions. The hyper-methylated regions were associated with 20 down- and 21 up-regulated genes, whereas the hypo-methylated regions included 21 down- and 41 up-regulated genes (Supplementary Table S10). Therefore, there was a negative correlation between the methylation level and transcript abundance for more than half of the DMR-associated DEGs. For example, some genes (e.g., *OsPRR9, OsCatalase-2*, and *OsWRKY11*) were more highly expressed in the hypo-methylated regions of DK151 plants than in the IR64 plants. Additionally, the expression levels of genes encoding a cysteine proteinase, histone, K^+^ eﬄux antiporter-2, a tetratricopeptide repeat-like superfamily protein, and zinc transporter-11 were lower in the hyper-methylated regions of DK151 plants than in the IR64 plants under control conditions.

We also compared the drought-induced DMRs and DEGs from DK151 and IR64 lines. We determined that 12 of 92 DMRs were associated with 12 DEGs in DK151 plants (Supplementary Table S11), while 23 of 506 DMRs were associated with 23 DEGs in IR64 plants (Supplementary Table S12). None of the DMR-associated genes were detected in both genotypes. Genes encoding the LEA protein, NAC TF, Osmotin-34, and leucine-rich receptor-like protein kinase were demethylated, but their expression levels were significantly up-regulated in drought-stressed DK151 plants. Additionally, genes encoding the oxysterol-binding protein and an expressed protein were hyper-methylated, while their expression levels were down-regulated in drought-stressed DK151 plants. Genes encoding POZ/BTB-containing G-protein-1, a protein phosphatase 2C family protein, an NB-ARC domain-containing disease resistance protein, trehalose phosphate synthase, a MYB TF, and prefold in subunit 3 were demethylated in IR64 plants exposed to drought conditions, but their expression levels were up-regulated. Three genes encoding proteins with unknown functions were hyper-methylated in drought-stressed IR64 plants, while their expression levels were down-regulated. The remaining DMR-associated genes in the two genotypes exhibited a positive correlation between the methylation level and transcript abundance. Overall, our results indicate that the relationship between DNA methylation and gene expression is complex.

## Discussion

### Methylated DNA Immunoprecipitation Sequencing Analysis of Two Contrasting Drought-Tolerant Genotypes

DNA methylation has been widely implicated in physiological and developmental processes in plants. With the development of next-generation sequencing technology, several genome-wide DNA methylation profiling platforms are now viable options, including bisulfite sequencing and MeDIP-seq. Bisulfite sequencing is still considered the gold standard for profiling DNA methylation at a single base pair resolution (Li et al., 2010; Taiwo et al., 2012; Crampton et al., 2016). However, MeDIP-seq is a high-throughput and more affordable method that may be appropriate for routine use, even though the coverage and resolution are lower. In this study, we used MeDIP-seq to profile the DNA methylation status of a drought-tolerant introgression rice line (DK151) and its recurrent parent (IR64) under drought and control conditions. The MeDIP-seq data indicated that DNA methylation is more common in the gene body regions than in the other gene regions. This result is consistent with those of a previous study (Zhang et al., 2006). Analyses of methylation peaks revealed that more than one-third of the peaks are located in intergenic regions, which is similar to the methylation peak distribution pattern in the *Arabidopsis thaliana* (Yong-Villalobos et al., 2015) and poplar genomes (Vining et al., 2012).

### Introgression and DNA Methylation Changes

Alien introgressions may result in genome-wide DNA methylation changes in animals (Müller et al., 2001) and plants (Liu et al., 2004; Dong et al., 2006). In this study, more DMRs were detected in the introgression line DK151 than in its recurrent parent IR64 under normal growth conditions. These DMRs were evenly distributed among 12 chromosomes. Only 125 of 1,190 DMRs overlapped with introgressed regions, indicating that the introgression of donor genomic DNA induces genome-wide DNA methylation changes. The extensive epigenetic effects of alien introgressions may result from a general epigenetic disturbance, the introduction of exogenous methylation-modifying factors from the donor, or genomic rearrangements (Müller et al., 2001; Riddle and Richards, 2002; Dong et al., 2006). However, the molecular mechanisms regulating these considerable DNA methylation changes require further study.

There is evidence indicating that altered DNA methylation induced by alien chromosomal segments may have contributed to the development of abiotic stress tolerance (Wang M. et al., 2014). More interestingly, GO enrichment analyses revealed that most of the DMR-associated genes in DK151 are related to stress responses, programmed cell death, nutrient reservoir activity, lyase activity, and magnesium ion binding. This observation implies that introgression-induced changes to the DNA methylation status of these genes may be correlated with the constitutive drought tolerance of DK151 plants.

### Functions of DNA Methylation in Drought Stress Tolerance

The extent and pattern of DNA methylation differ among diverse rice genotypes with various stress tolerance phenotypes (Zheng et al., 2013; Garg et al., 2015). There is also evidence that genome-wide DNA methylation changes are involved in abiotic stress responses in plants (Eichten and Springer, 2015). In this study, numerous DMRs were detected in both drought-stressed genotypes, indicating that drought-induced DNA methylation changes have a considerable impact on drought stress responses in rice plants. There were fewer drought-induced DMRs detected in DK151 plants than in IR64 plants, which further confirms the results of a previous study that concluded that the methylome is more stable in a drought-tolerant genotype than in a drought-sensitive genotype during exposures to drought conditions (Zheng et al., 2013).

Gene ontology analyses of DMR-associated genes in DK151 plants under drought conditions revealed that the hypo- or hyper-methylated genes are mainly related to transport, stress responses, and transcription regulation. Additionally, several TF genes, including those encoding WRKY, TCP, NAC, ARF, and AP2, were uniquely differentially methylated in DK151 plants under drought stress. This implies that drought-induced DNA methylation changes may regulate the expression of genes associated with drought stress responses by affecting TF genes. Our results are consistent with those of a previous study that revealed stress-induced DNA methylation changes of TF genes are repressed or activated in soybean plants under salt stress conditions (Song et al., 2012). Two cell wall-associated kinase genes (*OsWAK2* and *OsWAK3*) and two *Osmotin-34* genes were only hypo-methylated in DK151 under drought conditions. These genes influence stress responses (Zhang et al., 2005; de Oliveira et al., 2014), and the decreased DNA methylation may positively affect the drought tolerance of DK151 plants.

The genes encoding NTF2, vacuolar protein sorting 46.1, and hydrophobic protein LTI6A exhibited contrasting DNA methylation patterns in DK151 and IR64 plants under drought conditions. The NTF2 protein is essential for nuclear trafficking, and is involved in importing proteins into the nucleus (Zhao et al., 2006). The expression of *NTF2* was up-regulated by drought independently of abscisic acid (Hu et al., 2011). Vacuolar protein sorting 46.1 is an endosomal sorting complex required for transport-related protein, and it affects intracellular protein trafficking (Spitzer et al., 2009). Hydrophobic protein LTI6A is a transmembrane protein that may influence transport (Rudd et al., 1998). Differences in the changes to the DNA methylation of these three genes imply that the transport of biomacromolecules in DK151 and IR64 plants under drought conditions is regulated by epigenetic remodeling. These observations suggest that DNA methylation changes influence drought stress responses by epigenetically regulating genes with diverse functions.

### DNA Methylation has Diverse Effects on Gene Expression

The regulatory effects of DNA methylation on plant gene expression have been widely studied. Methylation of the promoter region generally represses gene expression while the methylation of the gene body has the opposite effect (Zilberman et al., 2007; Li et al., 2012). In this study, we compared the methylome and transcriptome of two rice genotypes with contrasting drought tolerance phenotypes. Our results revealed that the relationship between DNA methylation status and gene expression level is more complex than expected. First, we detected 1,190 DMRs and 5,641 DEGs between DK151 and IR64 under normal growth conditions. Only 103 DEGs were located in 41 hyper- and 62 hypo-methylated DMRs, indicating that a small proportion of the DMR-associated genes exhibit a significantly different expression level between the two genotypes. The same trend was observed for the drought-induced DMRs and DEGs for both genotypes, with only a few DMR-associated genes differentially expressed between the drought-stressed and control plants. These results are consistent with those of several previous studies that revealed that in most cases, differences in gene expression levels are not correlated with differences in DNA methylation (Garg et al., 2015; Secco et al., 2015; Wang et al., 2015). The effects of DNA methylation on gene expression may be directly or indirectly mediated through common or unique transcriptional pathways.

Second, we observed positive and negative correlations between DNA methylation and gene expression. A negative correlation between methylation level and transcript abundance was detected for more than half of the DMR-associated genes, which is in agreement with the findings of several previous studies (Zilberman et al., 2007; Li et al., 2012; Vining et al., 2012; Hu et al., 2015). However, we identified many DK151 and IR64 genes for which there was a positive relationship between DNA methylation and transcript abundance under control and drought stress conditions. The DMRs in different gene regions have diverse effects on gene expression (Liang et al., 2011; Dubin et al., 2015), indicating there is no direct linear relationship between the methylome and transcriptome. Furthermore, the effects of DNA methylation on plant gene expression were observed to be tissue/organ-specific (Gutierrez-Arcelus et al., 2015) and dependent on the developmental stage (Wang et al., 2015; Yong-Villalobos et al., 2015). Specifically, the DNA methylation changes in drought-stressed plants occurred primarily in genes that encoded proteins with unique functions. This is consistent with the results of earlier studies (Zilberman et al., 2007; Wang J. et al., 2014; Colicchio et al., 2015). Therefore, DNA methylation has diverse effects on gene expression, and the relationship between methylation status and expression is more complicated than expected.

## Conclusion

We detected genome-wide differences in DNA methylation between the drought-tolerant introgression line DK151 and its drought-sensitive recurrent parent IR64 under drought and control conditions. The introgression induced global DNA methylation changes in DK151 are implicated to its constitutive drought tolerance. And drought stress induced differential DNA methylation alterations in two genotypes, the DMR-associated genes are involved in genotype-specific drought responses. A comparison of the methylome and transcriptome revealed that the relationship between DNA methylation and gene expression is complex. Some genes that were differentially methylated exhibited significantly altered gene expression, implying they may be involved in the molecular regulation of drought stress responses.

## Author Contributions

WW and QQ performed the DNA methylation and transcriptome analyses, FS, YW and DX performed sampling and bisulfate sequencing analyses, ZL and BF interpreted data and wrote the manuscript.

## Conflict of Interest Statement

The authors declare that the research was conducted in the absence of any commercial or financial relationships that could be construed as a potential conflict of interest.

## Footnotes

1 http://broadinstitute.github.io/picard/

2 http://katahdin.mssm.edu/kismeth/revpage.pl

3 http://frodo.wi.mit.edu/
