# Supplementary material for: Genome-Wide Differences in DNA Methylation Changes in Two Contrasting Rice Genotypes in Response to Drought Conditions
Source: Front Plant Sci. 2016 Nov 8;7:1675. doi: 10.3389/fpls.2016.01675 (PMC5099141; doi:10.3389/fpls.2016.01675)
Supplement: Supplementary file 13 [file Data_Sheet_13.PDF]

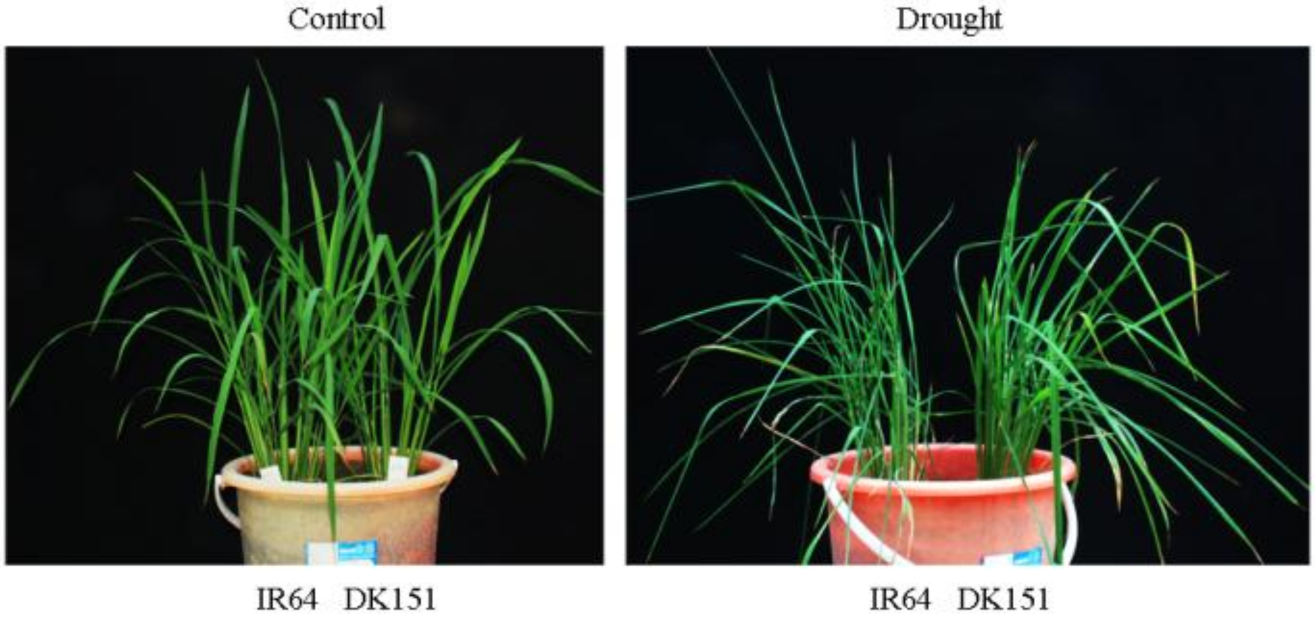

Figure S1. Phenotypic differences between IR64 and DK151 under control and drought conditions.

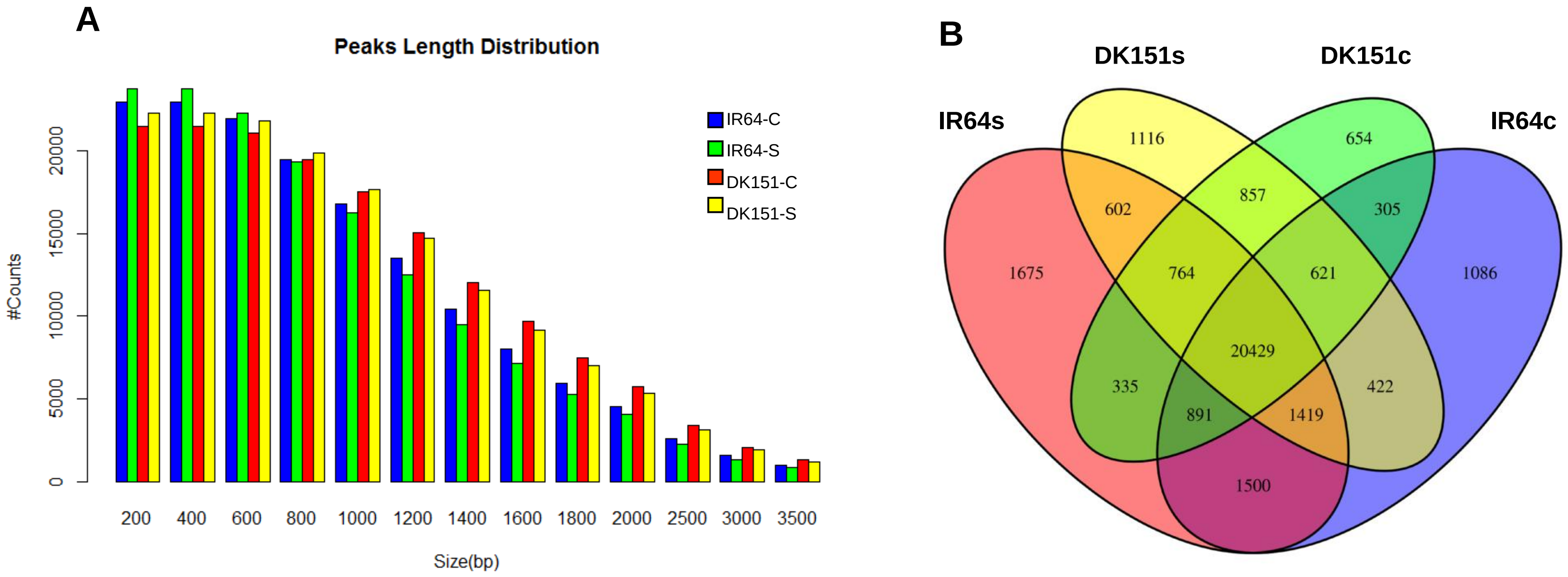

Figure S2. Peaks length distribution (A) and peak comparison by Venn diagram results (B) in IR64 and DK151 under drought stress (s) and control (c) conditions.

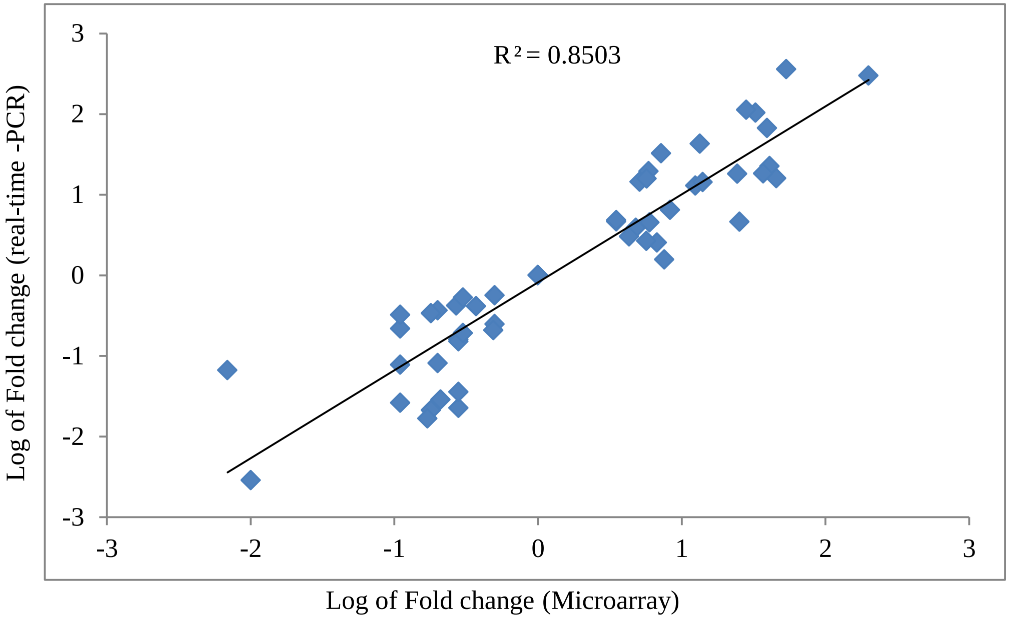

Figure S3. Validation of the expression of 49 random selected genes by qRT-PCR. Correlation analysis shows a good agreement between microarray and qRT-RCR experiments. The fold change values were transformed to log scale.
